# Hierarchical Attention Neural Network for Event Types to Improve Event Detection

**DOI:** 10.3390/s22114202

**Published:** 2022-05-31

**Authors:** Yanliang Jin, Jinjin Ye, Liquan Shen, Yong Xiong, Lele Fan, Qingfu Zang

**Affiliations:** 1 Key Laboratory of Specialty Fiber Optics and Optical Access Networks, Joint International Research Laboratory of Specialty Fiber Optics and Advanced Communication, Shanghai University, Shanghai 201620, China; stunning@shu.edu.cn (J.Y.); jsslq@shu.edu.cn (L.S.); jacfan@shu.edu.cn (L.F.); zangqingfu@shu.edu.cn (Q.Z.); 2 Shanghai Institute of Microsystem and Information Technology, Chinese Academy of Sciences, Shanghai 200050, China; yong.xiong@mail.sim.ac.cn

**Keywords:** event detection, LSTM, attention aggregation mechanism, neural module network

## Abstract

Event detection is an important task in the field of natural language processing, which aims to detect trigger words in a sentence and classify them into specific event types. Event detection tasks suffer from data sparsity and event instances imbalance problems in small-scale datasets. For this reason, the correlation information of event types can be used to alleviate the above problems. In this paper, we design a Hierarchical Attention Neural Network for Event Types (HANN-ET). Specifically, we select Long Short-Term Memory (LSTM) as the semantic encoder and utilize dynamic multi-pooling and the Graph Attention Network (GAT) to enrich the sentence feature. Meanwhile, we build several upper-level event type modules and employ a weighted attention aggregation mechanism to integrate these modules to obtain the correlation event type information. Each upper-level module is completed by a Neural Module Network (NMNs), event types within the same upper-level module can share information, and an attention aggregation mechanism can provide effective bias scores for the trigger word classifier. We conduct extensive experiments on the ACE2005 and the MAVEN datasets, and the results show that our approach outperforms previous state-of-the-art methods and achieves the competitive F1 scores of 78.9% on the ACE2005 dataset and 68.8% on the MAVEN dataset.

## 1. Introduction

**Figure 1:**
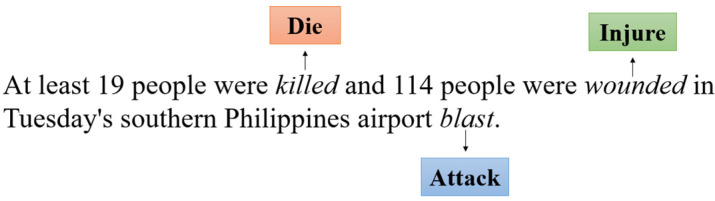
An example sentence of the ACE2005 dataset.

Event detection is a crucial subtask of event extraction, which aims to identify event trigger words and classify the corresponding event types from plain texts. Specifically, each event is often labeled by a word or phrase called an event trigger word. In event detection, one sentence may contain two or more trigger words, and these words will trigger a variety of event types. As shown in Figure 1, the event detection task should identify these trigger words “*killed*”, “*wounded*”, “*blast*” and classify them to the event types ***Die***, ***Injured***, ***Attack***, respectively.

Currently, the datasets used for event detection are limited. Due to the fact that event instance annotation is expensive and complex, most of the existing datasets are small-scale, which suffer from data sparsity and event instance imbalance problems. For example, the most widely-used ACE2005 English dataset only contains 599 documents, and 20 of its 33 event types have no more than 100 labeled instances, so the unbalanced event instances problem has a great influence on the effect of the event detection task, other small datasets have similar problems [1]. However, correlation information between various event types in the sentence can be utilized to alleviate the above problems. For instance, in Figure 1, from the prior knowledge, we can know that the event types ***Die***, ***Injure***, and ***Attack*** are more likely to appear together in a sentence, whereas ***Attack*** and ***Marry*** are less likely to occur at the same time. Moreover, take the following two sentences as examples:

***S1**: He **left** the company.*
***S2**: He **left** the company, and planned to **go** home directly.*

Sentence S1 only has the trigger word “*left*”, which may trigger ***End-Position*** and ***Transport***, two different event types. ***End-Position*** means he resigned from the company, while ***Transport*** means he stayed away from the company. Meanwhile, in sentence S2, we consider the trigger word “*go*” and can easily argue that word “*left*” triggers the ***Transport*** event. There are some approaches that utilize the aforementioned information to improve the performance of event detection tasks. Liao et al. [2] proposed a document-level statistical model to achieve document-level within-event and cross-event consistency. Liu et al. [3] proposed an approach that represented global information in the form of logic using a probabilistic soft logic model. Li et al. [4] proposed a joint framework that extracted triggers and arguments together to improve the performance. These methods dealt with the correlation information of various event types independently and could not tackle the data sparsity and event instance imbalance. Liu et al. [5] employed Type Group Regularization to obtain information between similar event types to alleviate the above problems, and their method was effective on the small-scale ACE2005 dataset. Deng et al. [6] proposed a novel ontology-based framework that enriched event ontology with event–event relation and induced more event correlations based on existing ones, and the experimental results on their handcrafted dataset showed great performance of their method.

In this paper, the hierarchical event type information is used to alleviate the above problems. Specifically, we define every event supertype as an upper-level module and divide several event subtypes that are more closely related in text into one upper-level module. Here we refer to [5] and divide the subtypes ***Die***, ***Injure***, ***Attack***, and ***Demonstrate*** into the same upper-level event type ***Conflict***, while the others stay the same as the ACE2005 dataset. We propose a novel model named Hierarchical Attention Neural Network for Event Types, abbreviated to HANN-ET. Inspired by previous work on hierarchical modules [7,8], we use Neural Module Networks (NMNs) [9] to construct hierarchical event type modules. Considering that the features of various supertypes have different weighted influences on trigger words in the sentence, we use the attention mechanism [10,11] to calculate the weighted influence of each upper-level module on trigger words; then we adopt a weighted attention aggregation mechanism to integrate each upper-level module attention score to acquire the correlation information of the event type. In the above, subtypes divided into the same upper-level module can share information, and every upper-level module provides an effective bias score for the trigger classifier through the attention mechanism. After that, we get the whole upper-level event type feature representation by employing an attention aggregation mechanism. In this way, our method effectively alleviates the problem of event instance imbalance in datasets. Moreover, we use the Graph Attention Network (GAT) [12] to get the syntactic representation for each word in the sentence, then we integrate it to enrich the whole representation of input text to improve the performance of event detection. In summary, the contributions of this paper are as follows.

1. We propose a novel network model called HANN-ET to alleviate data sparsity and event instance imbalance problems without using external resources in small-scale datasets. It also works well on large-scale datasets.
2. We employ a weighted attention aggregation mechanism instead of an average operation to merge the representations of all the upper-level event type modules, and we integrate syntactic information obtained by GAT to enrich the text representation.
3. We conduct experiments on the widely used small-scale ACE2005 and large-scale MAVEN datasets. The experimental results on both datasets demonstrate that our approach is effective for event detection tasks and achieves state-of-the-art performance.

## 2. Related Work

In early event detection tasks, researchers mainly use domain knowledge [13] to manually design language features, which is labor-intensive and requires external resources. Later, with the development of machine learning, the Hidden Markov Model (HMM) [14] and Conditional Random Field (CRF) [15] are utilized for event detection tasks. These models require the training of large-scale corpus, and the performance relies heavily on feature selection.

In recent years, most works have focused on deep learning [16] for event detection tasks. These works mainly exploit various neural networks such as Convolutional Neural Network (CNN) [17] and Long Short-Term Memory (LSTM) [18,19] to represent semantic vectors of text. In the last three years, syntactic relation representation has been proven to be useful for event detection tasks, and [20,21,22,23] employed the Graph Convolutional Network (GCN) to represent the features of syntactic dependence in a sentence. The authors of [24,25] used a variant of Recurrent Neural Network (RNN) to obtain not only sentence-level features but also document-level features to enrich the contextual information. Ngo N. T et al. [26] employ the Gumbel-Softmax method [27] to learn important or relevant words in the overall representation vector to benefit the task. These approaches all improve the performance of event detection. However, the above methods do not consider the data sparsity and event instances imbalance problem and ignore the correlation information and different weighted influence of event types in a sentence.

Deng et al. [28] and Lai et al. [29] proposed few-shot learning to alleviate the problem of data sparsity in small-scale datasets, which is a different and effective way to improve the performance of event detection. Wang et al. [30] employed BERT [31] as the sentence encoder and proposes an adversarial imitation model to expand more training data for the task. Wang et al. [32] improved Pre-trained Language Models (PLMs) to better utilize rich event knowledge in large-scale unsupervised data. They adopted the New York Times Corpus (NYT) [33] as the unsupervised pre-training corpora and used its raw text to build the AMR structures with a state-of-the-art AMR parser [34]. Tong et al. [35] built an image dataset supplement and conducted deep interactions between images and sentences for modality features aggregation. The authors of [2,3,4] considered the association information between event types, but they tackled various event types independently. Liu et al. [5] exploited Type Group Regularization to get correlation information between event types and help sparse types benefit from tense types. Their approach is useful, but they only conducted experiments on the small-scale ACE2005 dataset. Deng et al. [6] built event ontology embedding through BERT and designed an event correlation inference mechanism to get new event correlations based on symbolic rules. Then, they created a novel dataset with event correlations based on two newly proposed datasets: MAVEN [1] and FewEvent [26]. The results from the created dataset proved the effectiveness of their approach. In our method, the proposed upper-level modules use correlation information between event types through Neural Module Networks, and the experimental results on two different datasets demonstrate that our model successfully improves the performance of the event detection task.

## 3. Our Model

**Figure 2:**
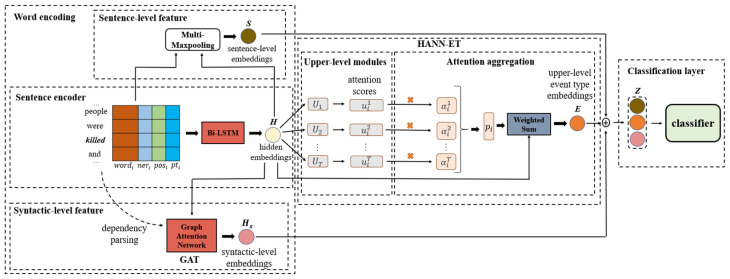
The overall architecture of HANN-ET.

In this section, we will introduce the proposed model. Shown in Figure 2 is the overall architecture of HANN-ET. Our model is mainly composed of three components:

1. Word encoding: we first represent a sentence into hidden embeddings via the LSTM model, then we use the dynamic multi-pooling to aggregate sentence information into sentence-level embeddings. Meanwhile, we utilize GAT to get syntactic-level embeddings.
2. HANN-ET: we adopt Neural Module Networks to build the weighted scores about the upper-level modules of the event types, then we employ the attention mechanism to aggregate the scores from several upper-level modules, finally, we calculate the weighted sum of hidden embeddings as upper-level event type embeddings.
3. Classification layer: we rely on sentence-level embeddings, syntactic-level embeddings, and upper-level event type embeddings to estimate the probability of a specific event type for the sentence.

### 3.1. Word Encoding

Same as the existing works, we regard event detection as a sequence labeling task. Consider that the trigger words in the sentence may contain multiple words, so we adopt the “BIO” schema to make an annotation. The event type information is obtained from a predefined set of events. Thus, the total number of labels is 2×L+1, 2 for “B” and “I”, 1 for “O”, and L is the number of predefined event types. Let $X=x_{1},x_{2},\ldots ,x_{n}$ be a sentence of length n, where $x_{i}$ is its i-th token.

#### 3.1.1. Sentence Encoder

In the sentence encoder component, we first transform each input token $x_{\dot{i}}\in X$ into a real-valued embedding vector $w_{i}$. Following the previous work on event detection [24,25], the comprehensive embedding vector $w_{i}$ is concatenated with its word embedding $word_{i}$, entity type embedding $ner_{i}$, POS-tagging embedding $pos_{i}$, and position embedding $pt_{i}$, where $word_{i}$ is represented by looking up a pre-trained word embedding table on a large corpus and others are randomly initialized. Here,

(1) $$w_{i}=word_{i}⊕ner_{i}⊕pos_{i}⊕pt_{i}$$

where ⊕ represents the concatenation operation.

After the transformation of token $x_{\dot{i}}$ to vector $w_{i}$, we get a sequence of vectors $W=w_{1},w_{2},\ldots ,w_{n}$ for the input sentence X. Then, we employ a Bidirectional Long-Short Term Memory network (Bi-LSTM) [20] as the sentence encoder to encode W and incorporate the sentence context information into the representation vector $H=h_{1},h_{2},\ldots ,h_{n}$. It will be used as the input of the HANN-ET and GAT. $h_{i}$ is obtained from the following formula:

(2) $$h_{i}=\left[\vec{LSTM}\left(w_{i}\right)⊕\overset{\leftarrow }{LSTM}\left(w_{i}\right)\right]$$

where $\vec{LSTM}\left(w_{i}\right)$ and $\overset{\leftarrow }{LSTM}\left(w_{i}\right)$ are the hidden states of the forward and backward LSTM at position i, respectively.

#### 3.1.2. Sentence-Level Feature

As one sentence may contain two or more events, we make use of a dynamic multi-pooling layer [17] for Bi-LSTM to get sentence-level embeddings $S=s_{1},s_{2},\ldots ,s_{n}$. We split each dimension of H into two parts according to the candidate triggers. The dynamic multi-pooling is represented as follows:

(3) $${\left[s_{1,p_{t}}\right]}_{j}=max\left\{{\left[h_{1}\right]}_{j},\ldots ,{\left[h_{p_{t}}\right]}_{j}\right\}$$

(4) $${\left[s_{p_{t}+1,n}\right]}_{j}=max\left\{{\left[h_{p_{t}+1}\right]}_{j},\ldots ,{\left[h_{n}\right]}_{j}\right\}$$

(5) $$s=\left[s_{1,p_{t}}\right]⊕\left[s_{p_{t}+1,n}\right]$$

where ${\left[\cdot \right]}_{j}$ is the j-th dimension of the vector, and $p_{t}$ is the position of the candidate trigger. We concatenate the two max-pooling results to represent a sentence-level feature.

#### 3.1.3. Syntactic-Level Feature

To get a syntactic-level feature, we follow the existing work [25], which employs a multi-order Graph Attention Network [11] to weigh the importance of neighbors of each word in each syntactic graph network. We denote the first-order syntactic graph with adjacency matrix A, which contains three sub matrices $A_{along}$, $A_{rev}$, $A_{loop}$ with the same dimensions n×n. Then the k-th order syntactic graph is described as $A_{subg}^{k}$, where $subg\in \left\{along,rev,loop\right\}$, $A_{rev}^{k}={\left(A_{along}^{k}\right)}^{⊤}$. $A_{loop}^{k}$ is the identity matrix. If there is a dependency arc from word $x_{i}$ to $x_{j}$ in the dependency tree, then $A_{along}^{k}\left(i,j\right)=1$, otherwise 0. We obtain the representation $h_{i}^{k}$ of the k-th-order syntactic graph $A^{k}$ by the following formulas,

(6) $$h_{i}^{k}=GAT\left(h_{i},A_{along}^{k}\right)+GAT\left(h_{i},A_{rev}^{k}\right)+GAT\left(h_{i},A_{loop}^{k}\right)$$

(7) $$GAT\left(h_{i},A_{along}^{k}\right)=\sigma \overset{n}{\underset{j=1}{\sum }}\left(u_{ij}A_{\left(along\right)ij}^{k}\left(W_{along,k}h_{i}+\epsilon_{along,k}\right)\right)$$

(8) $$u_{ij}=\frac{exp\left(leakyRelu\left(W_{c}\left[W_{att}h_{i}⊕W_{att}h_{j}\right]\right)\right)}{\sum_{j\in N_{i}}exp\left(leakyRelu\left(W_{c}\left[W_{att}h_{i}⊕W_{att}h_{j}\right]\right)\right)}$$

where + is element-wise addition in Equation (6). σ in Equation (7) is the exponential linear unit, $W_{along,k}$ and $\epsilon_{along,k}$ are the weight matrix and bias item, respectively. In Equation (8), $u_{ij}$ is a normalized weight that indicates the importance of word $x_{j}$ to $x_{i}$, and $N_{i}$ is some neighborhood of $x_{i}$ in the subgraph. According to Veličković et al. [12], the negative input slope α of the LeakyRelu function is set to 0.2, and $W_{c}$ and $W_{att}$ are weight matrices [11].

Then, we employ the attention aggregation mechanism [36] to get the whole multi-order representations as syntactic-level embedding $H_{s}=h_{s1},h_{s2},\ldots ,h_{sn}$:

(9) $$s_{i}^{k}=tanh\left(W_{as}h_{i}^{k}+\epsilon_{as}\right)$$

(10) $$v_{i}^{k}=\frac{exp\left({\left(s_{i}^{k}\right)}^{⊤}u_{ctx}\right)}{\sum_{k=1}^{K}exp\left({\left(s_{i}^{k}\right)}^{⊤}u_{ctx}\right)}$$

(11) $$h_{si}=\overset{K}{\underset{k=1}{\sum }}v_{i}^{k}h_{i}^{k}$$

here $W_{as}$ and $\epsilon_{as}$ are the weight matrix and the bias term, respectively; $u_{ctx}$ is a randomly initialized vector that represents the influence of graph structure of each order.

### 3.2. HANN-ET

As shown in Figure 2, we build several upper-level event type modules through Neural Module Networks. Every upper-level module gives an attention score for each hidden embedding to represent the correlation with the specific upper-level module. Moreover, a trigger word may belong to more than one upper-level module, and every upper-level module does not equally relate to the trigger words. Hence, we employ a weighted attention aggregation mechanism to merge the scores from several upper-level modules together. Then, we compose all hidden embeddings with their corresponding attention scores as the whole upper-level event type feature. According to the properties of the ACE2005 dataset, we design eight upper-level modules for it. For the MAVEN dataset, [1] has shown the tree-structure hierarchical event type schema. Eventually, we build five upper-level event type modules.

#### 3.2.1. Upper-Level Modules

**Figure 3:**
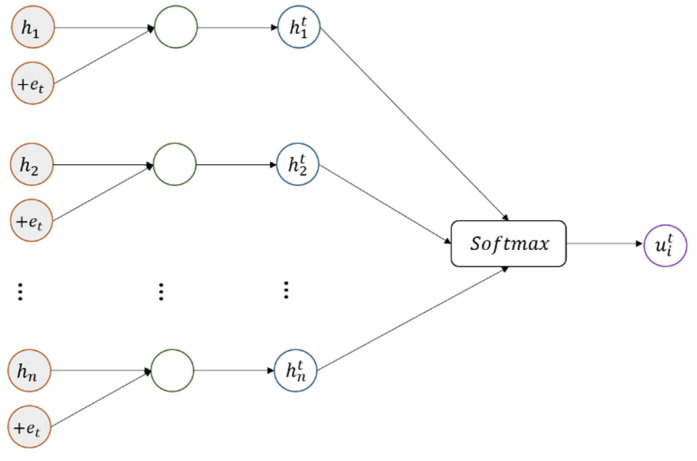
Attention score of the specific upper-level module.

As shown in Figure 3, following previous work [37], for a specific upper-level module t, we adopt a multi-layer perceptron to get $h_{i}^{t}$ as a hidden representation of $h_{i}$,

(12) $$h_{i}^{t}=tanh\left(W_{a}\left[h_{i}⊕e_{t}\right]\right)$$

where $W_{a}$ is the trainable weight matrix and $e_{t}$ is a trainable vector that represents the semantic feature of a specific upper-level module t. The initial representation of the vector $e_{t}$ is different for different upper-level modules. Then, for the hidden representation $h_{i}^{t}$, we get the attention score of the specific upper-level module t through a softmax function:

(13) $$u_{i}^{t}=\frac{\exp\left(W_{b}h_{i}^{t}\right)}{\sum_{j=1}^{n}\exp\left(W_{b}h_{j}^{t}\right)}$$

where $W_{b}$ is the trainable weight matrix.

#### 3.2.2. Attention Aggregation

The attention mechanism as an idea does not have a specific network structure for restriction [10]. It contains a general understanding of the global elements and captures the connections between the main elements. In the commonly used self-attention networks, the calculation formulas are as follows:

(14) $$Attention\left(Q,K,V\right)=softmax\left(\frac{Q\cdot K^{⊤}}{\sqrt{d_{k}}}\right)V$$

In Equation (14), Q, K, V are the inputs, and $d_{k}$ is the dimension of Q. The attention score represents the weight influence of V on the networks.

**Figure 4:**
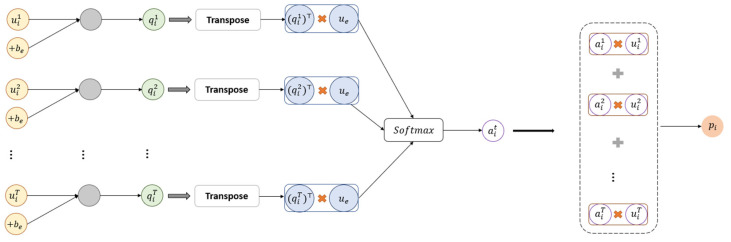
Attention weights for all upper-level modules.

As shown in Figure 4, inspired by the previous aggregation work [10,36], we use the attention mechanism to aggregate the scores from several upper-level modules. We first get $q_{i}^{t}$ as an attentional module representation of $u_{i}^{t}$ via multi-layer perceptron, then we measure the importance of the specific module and get a normalized importance weight $\alpha_{i}^{t}$ through a softmax function. Finally, we obtain the vector $p_{i}$ as a weighted sum of all modules based on the weights. The calculation formulas are as follows:

(15) $$q_{i}^{t}=tanh\left(W_{e}u_{i}^{t}+b_{e}\right)$$

(16) $$\alpha_{i}^{t}=\frac{exp\left({\left(q_{i}^{t}\right)}^{⊤}u_{e}\right)}{\sum_{j=1}^{T}(exp{\left(q_{i}^{j}\right)}^{⊤}u_{e})}$$

(17) $$p_{i}=\sum_{t=1}^{T}\alpha_{i}^{t}u_{i}^{t}$$

here $W_{e}$ and $b_{e}$ are the weight matrix and the bias term, respectively. In Equation (16), $u_{e}$ is a randomly initialized vector and jointly learns during the training process to measure the importance of event type representation of each upper-level module. T is the number of upper-level modules.

Finally, we calculate the weighted sum of hidden embeddings as the upper-level event type embeddings:

(18) $$E=\sum_{i=1}^{n}p_{i}h_{i}$$

### 3.3. Classification Layer

We concatenate sentence-level embeddings S, upper-level event type embeddings E, and syntactic-level embeddings $H_{s}$ into embeddings Z as the input for the classification layer. The layer is followed by a softmax function to estimate the probability for all event types of instance X:

(19) $$Z=S⊕E⊕H_{s}$$

(20) $$p\left(t|X\right)=\frac{\exp\left(W_{o}Z+\epsilon_{o}\right)}{\sum_{1}^{2L+1}exp\left(W_{o}Z+\epsilon_{o}\right)}$$

where $W_{o}$ and $\epsilon_{o}$ are the weight matrix and the bias term, respectively. After softmax classification, the event label with the largest probability is regarded as the result.

We exploit a bias loss function [25,30] to enhance the influence of event type labels during training since the number of “O” tags is much larger than that of event type tags. The loss function is defined as follows:

(21) $$J\left(\theta \right)=-\overset{N_{s}}{\underset{i=1}{\sum }}\overset{n_{i}}{\underset{j=1}{\sum }}logp\left(y_{j}^{t}|s_{i},\theta \right)\cdot I\left(O\right)+\mu log\left(y_{j}^{t}|s_{i},\theta \right)\cdot \left(1-I\left(O\right)\right)$$

where $N_{s}$ is the number of sentences, $n_{i}$ is the number of words in the i-th sentence, $I\left(O\right)=1$, if the tag is “O”, otherwise 0, μ is the bias weight.

## 4. Experiments

### 4.1. Experimental Setting

#### 4.1.1. Datasets and Evaluation Metrics

We utilize the ACE2005 and the MAVEN corpus as our datasets. The ACE2005 is the most widely-used dataset, which contains 599 documents, 5349 annotated instances, and 33 event types. It is a small-scale dataset. On the contrary, the MAVEN is a comprehensive and large dataset for event detection that contains 4480 documents, 118732 event mention instances, and 168 event types. For a reasonable comparison, we perform experiments on both datasets separately on several modern baselines in two conditions: predefined split and cross-validation. In the case of predefined split, we employ the same data split in the ACE2005 dataset as existing work [17,18,22,25], where 40 newswire documents are used as the test set, the development set with 30 other documents randomly selected from different genres, and the other 529 documents are used for training. In the MAVEN dataset, we operate the same as [1], where 2913 documents are utilized as the training set, 710 documents for the development set, and 857 documents for the test set. In a cross-validation situation, we divide the ACE2005 dataset equally into 15 parts; each part contains about 40 documents. The MAVEN dataset is divided equally into 5 parts, and each part has 896 documents. We exploit the Stanford CoreNLP toolkit for sentence splitting, tokenizing, POS-tagging, and dependency parsing. We use Precision (*P*), Recall (*R*), and *F*_1_-score as the evaluation metrics. The formulas are as follows.

(22) $$P=\frac{TP}{TP+FP}$$

(23) $$R=\frac{TP}{TP+FN}$$

(24) $$F_{1}=\frac{2PR}{P+R}$$

where *TP*, *TN*, *FP*, and *FN* denote the true positive, true negative, false positive, and false negative cases of the prediction results, respectively. The information about the machine in use to train the model is shown in Table 1.

#### 4.1.2. Hyper-Parameter Setting

We make our parameter selection according to the performance of the development set of datasets and previous work. The word embedding is obtained by the Word2Vec model, while entity type embedding, POS-tagging embedding, and position embedding are generated by looking up the randomly initialized embedding table. We set the word embedding size to 100 dimensions and the rest embedding size to 50 dimensions [21]. The hidden state size of the Bi-LSTM network is set to 100. According to Luong et al. [37], the semantic vector of upper-level module $e_{t}$ and trainable matrix $W_{b}$ are set to 900 and 900 dimensions, respectively. We set the highest order K to 3 and the dimension of graph feature to 150 [12]. We fix the maximum input sentence length n to 50 by padding shorter sentences and cutting longer ones. We set the batch size to 30 and exploit AdaDelta as the model optimization with an initial learning rate of 0.001 [25]. We set the dropout rate to 0.5 and the L2-norm regularization to 1 × 10^−5^ to avoid overfitting [23]. The bias loss parameter μ is set to 1. The values of hyper-parameters are shown in Table 2.

### 4.2. Overall Performance 

We use the modern baselines below for the predefined split and cross-validation experiments on two datasets and compare them with our method:

(1) CRF [15], a traditional machine learning method, views the event detection task as a sequence labeling problem for trigger words; the candidate events are obtained based on candidate trigger words which are identified through dictionary marching on the split sentences.
(2) DMCNN [17] builds a dynamic multi-pooling CNN model to learn sentence features. It uses CNN for basic feature extraction, and in the trigger classification stage, dynamic multi-pooling is proposed to split the feature map into two parts according to the candidate trigger, by which the most important features of each part can be obtained.
(3) JRNN [19] employs a bidirectional RNN as a feature extractor for the joint event extraction task, including event detection and arguments classification. It proposes a memory matrix that can effectively capture the dependencies between argument roles and trigger subtypes.
(4) HBTNGMA [25] fuses sentence-level and document-level information to enhance the semantic features. First, it exploits a hierarchical and bias tagging network to capture event interdependency and detect multiple events in one sentence collectively; then it devises a gated multi-level attention mechanism to automatically extract and integrate contextual information.
(5) JMEE [21] utilizes the self-attention and highway network to enhance GCN for event detection. It employs a syntactic Graph Convolution Network module to perform feature extraction by introducing shortcut arcs from syntactic structures. In the trigger classification module, a self-attention mechanism is added to capture the associations between multiple events in a sentence.
(6) AD-DMBERT [30] proposes an adversarial imitation model to expand more training data for the task. It creates a large event-related candidate set based on the ACE2005 dataset and then applies an adversarial training mechanism to iteratively identify those informative instances from the candidate set. It selects CNN and BERT as representative encoders to encode the given instances.
(7) MOGANED [22] improves GCN by combining multi-order word representation from different GAT layers. It uses Bi-LSTM to encode the input sentence to a sequence of vectors and proposes a multi-order Graph Attention Network that performs graph attention convolution over multi-order syntactic graphs. After that, it exploits an attention mechanism to aggregate multi-order representations of each word to predict its label.
(8) EE-GCN [23] proposes a novel architecture to use dependency label information, which conveys rich and useful linguistic knowledge for event detection tasks. It designs an edge-aware node update module that aggregates syntactically connected words through specific dependency types to generate expressive word representations. Furthermore, it devises a node-aware edge update module to refine the relation representations with contextual information.
(9) OntoED [6] links each event instance to a specific type in a target event ontology. It builds event ontology embedding through BERT and designs an event correlation inference mechanism to induce more event correlations based on existing ones. By the above, data-rich event types can propagate correlation knowledge to data-poor ones, and new event types can establish linkages to the event ontology.

Table 3 shows the overall performance of these baselines on two datasets in cases of predefined split. From the results, we have the following observations: (1) As compared with the basic models, DMCNN, JRNN, and HBTNGMA, HANN-ET achieves significant improvements on both datasets and achieves F1 scores of 77.6% and 65.6%, respectively. Furthermore, it outperforms or achieves competitive results with the JMEE, MOGANED, and EE-GCN, which also use dependency parsing information. This proves the effectiveness of our proposed upper-level modules and weighted attention. (2) MOGANED achieves the highest precision score of 79.5% but a lower recall score in the above models on the ACE2005 dataset; it is not hard to think that this is caused by the incorrect propagation from the dependency parsing tool. JMEE also suffers from this problem. In contrast, EE-GCN introduces dependency label information to capture more fine-grained trigger-related features, and HANN-ET builds the upper-level modules to obtain event type features; both models improve recall to some extent and achieve recall scores of 78.6% and 78.8%, respectively. (3) It is noteworthy that compared to all the baselines, HANN-ET[B] achieves the best recall and $F_{1}$ scores on the ACE2005 dataset of 79.6% and 78.9%, respectively, and the highest $F_{1}$ scores on the MAVEN dataset of 68.8%. Compared with HANN-ET, the recall scores of HANN-ET[B] on the ACE2005 and MAVEN datasets improved by 0.8% and 4.7%, respectively, the $F_{1}$ scores improved by 1.3% and 3.2%, respectively. It is not hard to analyze that the pre-training language model BERT learns event semantics and structure from large-scale unsupervised data and can significantly improve the recall score on the two datasets. (4) Compared with DMBERT and OntoED, which also adopt BERT to get more rich contextual features, HANN-ET[B] gets better $F_{1}$ scores on both datasets. (5) All models perform worse on the MAVEN dataset than on the ACE2005 dataset. In fact, larger datasets can lead to more robust performance of the models. Overall, our method utilizes correlation information between event types through neural module networks to improve the performance of the event detection task, and the competitive results demonstrate the effectiveness of our model.

Table 4 shows the overall performance of the models on the two datasets in the cross-validation case, and the overall results are similar to those in Table 3. From Table 4, we can see that HANN-ET outperforms or achieves competitive results with several models on two datasets, and HANN-ET[B] achieves the best performance, with $F_{1}$ scores of 78.6% and 69.2% on the two datasets, respectively. The cross-validation method is suitable for validating the performance of models on a small-scale dataset, such as the ACE2005. The experimental results demonstrate that our proposed model is effective and improves the performance of the event detection task in the cross-validation case.

### 4.3. Ablation Study

#### 4.3.1. The Validation of the Components

This section aims to demonstrate the effectiveness of upper-level event type modules, attention aggregation, and integrated feature models. To make the results of these methods below more robust, we conduct the ablation experiments in the case of cross-validation in the same way as Section 4.1.1 on the ACE2005 and MAVEN datasets. First, we designed several comparative architectures that have similar structures to our model to prove the validity of upper-level modules:

(1) DMCNN and HANN-ET-CNN, whereas the two models employ CNN as a sentence encoder and do not contain the GAT module.
(2) Bi-GRU (Gated Recurrent Unit) with a multi-pooling layer and HANN-ET-GRU, whereas the two models utilize GRU as a sentence encoder and do not contain the GAT module.
(3) Bi-LSTM with a multi-pooling layer and HANN-ET-LSTM, whereas the two models employ LSTM as a sentence encoder and do not contain the GAT module.
(4) To validate the impacts of attention aggregation and integrated feature model, we conduct an experiment on the HANN-ET-Mean model, which has the same modules as HANN-ET-LSTM but adopts mean operation to aggregate the attention scores.

As seen from Table 5, we can observe that: (1) As compared to three baseline extractors, the corresponding HANN-ET-based models achieve better performance on both datasets. The $F_{1}$ scores of the three HANN-ET-based models on the ACE2005 dataset improved by 1.1%, 2.2%, and 3.5%, respectively, and on the MAVEN dataset improved by 0.9%, 1.2%, and 2.5%. The results suggest that the upper-level event type modules have a great influence on event detection. (2) HANN-ET-Mean gets a lower performance than HANN-ET-LSTM, it means the attention aggregation mechanism is better than the average operation for aggregating upper-level module scores. HANN-ET, which integrates the syntactic representation via the GAT model, achieves the best performance in the above models and achieves $F_{1}$ scores of 77.1% and 66.1% in the two datasets, respectively; it indicates that the proposed integrated model can effectively improve the performance of the event detection task.

#### 4.3.2. The Experiments on General and Sparse Event Types

Our proposed model aims to utilize the correlation information between event types and provide effective bias scores through the attention mechanism to improve the performance on sparse event types. The small-scale ACE2005 dataset is suitable for this section of the experiment. We split the ACE2005 dataset into general and sparse groups based on the number of instances. According to our investigation, the number of instances of general and sparse event types in the most widely used ACE 2005 dataset is shown in Table 6. As seen in Table 6, there are 13 general subtypes and 20 sparse subtypes in the ACE2005 dataset. In sparse event types, the number of instances of each subtype is less than 100. We still employ cross-validation to conduct the experiments with several advanced baselines on general and sparse event types, respectively. Differently from Section 4.2, we also divide the ACE2005 dataset into 15 parts, but for general event types, the L in Equation (20) is set to 13. Similarly, the L is set to 20 for sparse event types.

The evaluation results are shown in Table 7. From the results, we have the following observations: (1) As compared with general subtypes, all the above methods achieve lower recall scores on the sparse event types since sparse subtypes contain few labeled instances in the ACE2005 dataset. (2) HANN-ET[B] achieves the best recall and $F_{1}$ scores with all the baselines on general and sparse subtypes when employing BERT to represent contextual word information, and HANN-ET achieves competitive results on general and sparse subtypes without extra resources. The results prove the effectiveness of our approach in tackling the sparse event types.

## 5. Conclusions

In this paper, we proposed a novel model named HANN-ET, which designed the upper-level modules to capture the correlation information of the event types. We considered three aspects at the sentence, syntactic, and upper-level event type, to enhance the overall semantic representation of the text. Our proposed model alleviates data sparsity and event instances imbalance problems in small-scale datasets for event detection tasks. Moreover, it does not depend on any external resources and improves the performance on large-scale datasets of event detection tasks. The experimental results on the ACE2005 and the MAVEN datasets show that our model is effective and achieves competitive performance on both datasets. The ablation study indicates the importance of the correlation information between the event types and the effectiveness of proposed upper-level modules. In the future, we will focus on improving the event detection task by Pre-trained Language Models, such as BERT, which uses a deep bidirectional Transformer structure to learn rich semantic representations from the large unlabeled corpus. Improvements to the Pre-trained Language Models are a trend for subsequent event detection tasks.

## Figures and Tables

**Table 1:** The experiment environment’s hardware and software.

Type	Configuration	Parameters
hardware	CPU	Intel(R) Core(TM) i7-10875H CPU @ 2.30GHz
	GPU	Nvidia GeForce RTX2060
	memory	16G DDR4 3200MHz
software	operating system	Windows 10
	compilation environment	Python 3.7
	deep learning framework	Tensorflow 1.13

**Table 2:** Hyper-parameter values.

Parameters	Values
word embedding dimension	100
entity type embedding dimension	50
POS-tagging embedding dimension	50
position embedding dim	50
LSTM hidden size	100
$e_{t}$ dimension	900
$W_{b}$ dimension	900
batch size	30
learning rate	0.001
dropout	0.5
regularization	1 × 10^−5^
μ	1

**Table 3:** Overall performance on two datasets in the case of predefined split (%).

Methods	ACE2005			MAVEN		
	*p*	R	$F_{1}$	*p*	R	$F_{1}$
CRF(2012)	65.3	59.7	62.4	53.8	52.4	53.1
DMCNN(2015)	75.6	63.6	69.1	**66.3**	55.9	60.6
JRNN(2016)	66.0	73.9	69.3	59.2	64.8	61.9
HBTNGMA(2018)	77.9	69.1	73.3	62.5	63.4	62.9
JMEE † (2018)	76.3	71.3	73.7	61.6	63.2	62.4
DMBERT(2019)[B]	77.9	72.5	75.1	62.7	**72.3**	67.1
MOGANED † (2019)	**79.5**	72.3	75.7	63.4	64.1	63.8
EE-GCN † (2020)	76.7	78.6	77.6	62.2	66.9	64.5
OntoED(2021) [B]	77.9	76.8	77.3	63.1	71.2	66.9
HANN-ET †	76.4	78.8	77.6	63.9	67.5	65.6
HANN-ET † [B]	78.3	**79.6**	**78.9**	65.7	72.2	**68.8**

† means the model is using a dependency structure, and [B] denotes a model adopting BERT as the instance encoder. **Bold** entries represent that the value is the highest result in the current comparative models.

**Table 4:** Overall performance on two datasets in the case of cross-validation (%).

Methods	ACE2005			MAVEN		
	*p*	R	$F_{1}$	*p*	R	$F_{1}$
CRF(2012)	65.6	59.2	62.2	54.5	51.8	53.1
DMCNN(2015)	75.8	65.2	70.1	65.8	57.2	61.2
JRNN(2016)	66.5	74.3	70.2	59.5	64.9	62.1
HBTNGMA(2018)	78.4	67.8	72.7	62.8	63.6	63.2
JMEE † (2018)	76.1	70.9	73.4	61.3	63.7	62.5
DMBERT(2019)[B]	78.2	73.7	75.9	62.5	**73.6**	67.6
MOGANED † (2019)	**79.8**	72.6	76.0	63.8	65.2	64.5
EE-GCN † (2020)	76.3	78.6	77.4	62.6	67.7	65.1
OntoED(2021)[B]	77.6	77.1	77.3	63.5	71.3	67.2
HANN-ET †	75.8	78.4	77.1	63.6	68.7	66.1
HANN-ET † [B]	77.9	**79.3**	**78.6**	**66.1**	72.6	**69.2**

† means the model is using a dependency structure, and [B] denotes a model adopting BERT as the instance encoder. **Bold** entries represent that the value is the highest result in the current comparative models.

**Table 5:** Results of ablation experiments on two datasets in the case of cross-validation (%).

Methods	ACE2005			MAVEN		
	*p*	R	$F_{1}$	*p*	R	$F_{1}$
DMCNN	**75.8**	65.2	70.1	**65.8**	57.2	61.2
HANN-ET-CNN	75.1	67.7	71.2	62.7	61.5	62.1
Bi-GRU-pooling	72.4	73.2	72.8	61.9	64.7	63.3
HANN-ET-GRU	73.9	76.2	75.0	62.6	66.5	64.5
Bi-LSTM-pooling	72.6	72.9	72.7	62.5	63.2	62.8
HANN-ET-LSTM	75.2	77.3	76.2	63.3	67.4	65.3
HANN-ET-Mean	73.5	76.0	74.7	62.1	66.1	64.0
HANN-ET	**75.8**	**78.4**	**77.1**	63.6	**68.7**	**66.1**

**Bold** entries represent that the value is the highest result in the current comparative models.

**Table 6:** The number of instances of general event types and sparse event types in the ACE2005 dataset.

	Subtype	Number
General event types	*Attack Transport Die Charge-Indict* *Meet End-Position Transfer-Money* *Elect Injure Transfer-Ownership* *Phone-Write Start-Position Trial-Hearing*	4460
Sparse event types	*Be-Born Marry Divorce Sue* *Start-Org Merge-Org Appeal Pardon* *End-Org Demonstrate Nominate* *Arrest-Jail Release-Parole Convict Fine* *Sentence Execute Extradite Acquit* *Declare-Bankruptcy*	889

**Table 7:** Results of experiments on general and sparse event types, respectively (%).

Methods	General Subtypes			Sparse Subtypes		
	*p*	R	$F_{1}$	*p*	R	$F_{1}$
DMCNN(2015)	87.5	80.3	83.7	89.2	46.2	60.9
JRNN(2016)	90.8	81.6	86.0	89.7	49.6	63.9
JMEE † (2018)	91.7	82.4	86.8	90.8	50.3	64.7
MOGANED(2019)	91.4	81.2	86.0	91.5	50.7	65.2
EE-GCN † (2020)	92.2	83.7	87.7	90.7	51.6	65.8
OntoED(2021)[B]	**93.6**	82.9	87.9	**92.3**	52.8	67.2
HANN-ET †	92.5	83.5	87.8	90.6	53.2	67.0
HANN-ET † [B]	93.1	**84.4**	**88.5**	91.4	**54.5**	**68.3**

† means the model is using a dependency structure, and [B] denotes a model adopting BERT as the instance encoder. **Bold** entries represent that the value is the highest result in the current comparative models.

## References

[1] Wang X., Wang Z., Han X., Jiang W., Han R., Liu Z., Zhou J. MAVEN: A Massive General Domain Event Detection Dataset. Proceedings of the 2020 Conference on Empirical Methods in Natural Language Processing.

[2] Liao S., Grishman R. Using document level cross-event inference to improve event extraction. Proceedings of the 48th Annual Meeting of the Association for Computational Linguistics.

[3] Liu S., Liu K., He S., Zhao J. A probabilistic soft logic based approach to exploiting latent and global information in event classification. Proceedings of the Thirtieth AAAI Conference on Artificial Intelligence.

[4] Li Q., Ji H., Huang L. Joint event extraction via structured prediction with global features. Proceedings of the 51st Annual Meeting of the Association for Computational Linguistics.

[5] Liu S., Chen Y., Liu K., Zhao J., Luo Z., Luo W. Improving event detection via information sharing among related event types. Proceedings of the Chinese Computational Linguistics and Natural Language Processing Based on Naturally Annotated Big Data.

[6] Deng S., Zhang N., Li L., Chen H., Tou H., Chen M., Huang F., Chen H. OntoED: Low-resource Event Detection with Ontology Embedding. Proceedings of the 59th Annual Meeting of the Association for Computational Linguistics and the 11th International Joint Conference on Natural Language Processing.

[7] Han X., Yu P., Liu Z., Sun M., Li P. Hierarchical relation extraction with coarse-to-fine grained attention. Proceedings of the 2018 Conference on Empirical Methods in Natural Language Processing.

[8] Wang X., Wang Z., Han X., Liu Z., Li J., Li P., Ren X. HMEAE: Hierarchical modular event argument extraction. Proceedings of the 2019 Conference on Empirical Methods in Natural Language Processing and the 9th International Joint Conference on Natural Language Processing.

[9] Andreas J., Rohrbach M., Darrell T., Klein D. Neural module networks. Proceedings of the IEEE Conference on Computer Vision and Pattern Recognition.

[10] Vaswani A., Shazeer N., Parmar N., Uszkoreit J., Jones L., Gomez A.N., Polosukhin I. (2017). Attention is all you need. Advances in Neural Information Processing Systems.

[11] Mehta S., Islam M.R., Rangwala H., Ramakrishnan N. Event detection using hierarchical multi-aspect attention. Proceedings of the World Wide Web Conference.

[12] Veličković P., Cucurull G., Casanova A., Romero A., Lio P., Bengio Y. Graph attention networks. Proceedings of the International Conference on Learning Representations.

[13] Ming L., Hailiang H. (2018). A method of extracting financial event information based on lexical-semantic model. Comput. Appl..

[14] Jiangde Y., Xinfeng X., Xiaozhong F. (2007). Chinese text event information extraction based on hidden Markov model. Microelectron. Comput..

[15] Hu B.L., He R.F., Sun H., Wang W.J. (2012). Chinese event type recognition based on conditional random field. Pattern Recognit. Artif. Intell..

[16] Pouyanfar S., Sadiq S., Yan Y., Tian H., Tao Y., Reyes M.P., Iyengar S.S. (2018). A survey on deep learning: Algorithms, techniques, and applications. ACM Comput. Surv. (CSUR).

[17] Chen Y., Xu L., Liu K., Zeng D., Zhao J. Event extraction via dynamic multi-pooling convolutional neural networks. Proceedings of the 53rd Annual Meeting of the Association for Computational Linguistics and the 7th International Joint Conference on Natural Language Processing.

[18] Hochreiter S., Schmidhuber J. (1997). Long short-term memory. Neural Comput..

[19] Nguyen T.H., Cho K., Grishman R. Joint event extraction via recurrent neural networks. Proceedings of the 2016 Conference of the North American Chapter of the Association for Computational Linguistics: Human Language Technologies.

[20] Nguyen T.H., Grishman R. Graph convolutional networks with argument-aware pooling for event detection. Proceedings of the Thirty-Second AAAI Conference on Artificial Intelligence.

[21] Liu X., Luo Z., Huang H. Jointly multiple events extraction via attention-based graph information aggregation. Proceedings of the 2018 Conference on Empirical Methods in Natural Language Processing.

[22] Yan H., Jin X., Meng X., Guo J., Cheng X. Event detection with multi-order graph convolution and aggregated attention. Proceedings of the 2019 Conference on Empirical Methods in Natural Language Processing and the 9th International Joint Conference on Natural Language Processing.

[23] Cui S., Yu B., Liu T., Zhang Z., Wang X., Shi J. Edge-Enhanced Graph Convolution Networks for Event Detection with Syntactic Relation. Proceedings of the 2020 Conference on Empirical Methods in Natural Language Processing.

[24] Zhao Y., Jin X., Wang Y., Cheng X. Document embedding enhanced event detection with hierarchical and supervised attention. Proceedings of the 56th Annual Meeting of the Association for Computational Linguistics.

[25] Chen Y., Yang H., Liu K., Zhao J., Jia Y. Collective event detection via a hierarchical and bias tagging networks with gated multi-level attention mechanisms. Proceedings of the 2018 Conference on Empirical Methods in Natural Language Processing.

[26] Ngo N.T., Nguyen T.N., Nguyen T.H. (2020). Learning to select important context words for event detection. Adv. Knowl. Discov. Data Min..

[27] Jang E., Gu S., Poole B. Categorical reparameterization with Gumbel-softmax. Proceedings of the International Conference on Learning Representations, Palais des Congrès Neptune.

[28] Deng S., Zhang N., Kang J., Zhang Y., Zhang W., Chen H. Meta-learning with dynamic-memory-based prototypical network for few-shot event detection. Proceedings of the 13th International Conference on Web Search and Data Mining.

[29] Lai V.D., Dernoncourt F., Nguyen T.H. (2020). Exploiting the matching information in the support set for few shot event classification. Adv. Knowl. Discov. Data Min..

[30] Wang X., Han X., Liu Z., Sun M., Li P. Adversarial training for weakly supervised event detection. Proceedings of the 2019 Conference of the North American Chapter of the Association for Computational Linguistics: Human Language Technologies.

[31] Jacob D., Chang M.-W., Lee K., Kristina T. BERT: Pre-training of Deep Bidirectional Transformers for Language Understanding. Proceedings of the 2019 Conference of the North American Chapter of the Association for Computational Linguistics: Human Language Technologies.

[32] Wang Z., Wang X., Han X., Lin Y., Hou L., Liu Z., Li P., Li J., Zhou J. CLEVE: Contrastive Pre-training for Event Extraction. Proceedings of the 59th Annual Meeting of the Association for Computational Linguistics and the 11th International Joint Conference on Natural Language Processing.

[33] Sandhaus E. (2008). The New York Times Annotated Corpus. Linguist. Data Consort..

[34] Xu D., Li J., Zhu M., Zhang M., Zhou G. Improving AMR Parsing with Sequence-to-Sequence Pre-training. Proceedings of the 2020 Conference on Empirical Methods in Natural Language Processing.

[35] Tong M., Wang S., Cao Y., Xu B., Li J., Hou L., Chua T.S. Image enhanced event detection in news articles. Proceedings of the AAAI Conference on Artificial Intelligence.

[36] Yang Z., Yang D., Dyer C., He X., Smola A., Hovy E. Hierarchical attention networks for document classification. Proceedings of the 2016 Conference of the North American Chapter of the Association for Computational Linguistics: Human Language Technologies.

[37] Luong M.T., Pham H., Manning C.D. Effective approaches to attention-based neural machine translation. Proceedings of the 2015 Conference on Empirical Methods in Natural Language Processing.

